# Response Inhibition Impairment in High Functioning Autism and Attention Deficit Hyperactivity Disorder: Evidence from Near-Infrared Spectroscopy Data

**DOI:** 10.1371/journal.pone.0046569

**Published:** 2012-10-09

**Authors:** Ting Xiao, Zhou Xiao, Xiaoyan Ke, Shanshan Hong, Hongyu Yang, Yanli Su, Kangkang Chu, Xiang Xiao, Jiying Shen, Yijun Liu

**Affiliations:** 1 Child Mental Health Research Center, Nanjing Brain Hospital affiliated of Nanjing Medical University, Nanjing, Jiangsu, China; 2 Key Laboratory of Child Development and Learning Science, Southeast University, Nanjing, Jiangsu, China; 3 Department of Psychiatry and McKnight Brain Institute, University of Florida, Gainesville, Florida, United States of America; University of Montreal, Canada

## Abstract

**Background:**

Response inhibition, an important domain of executive function (EF), involves the ability to suppress irrelevant or interfering information and impulses. Previous studies have shown impairment of response inhibition in high functioning autism (HFA) and attention deficit hyperactivity disorder (ADHD), but more recent findings have been inconsistent. To date, almost no studies have been conducted using functional imaging techniques to directly compare inhibitory control between children with HFA and those with ADHD.

**Method:**

Nineteen children with HFA, 16 age- and intelligence quotient (IQ)-matched children with ADHD, and 16 typically developing (TD) children were imaged using functional near-infrared spectroscopy (NIRS) while performing Go/No-go and Stroop tasks.

**Results:**

Compared with the TD group, children in both the HFA and ADHD groups took more time to respond during the No-go blocks, with reaction time longest for HFA and shortest for TD. Children in the HFA and ADHD groups also made a greater number of reaction errors in the No-go blocks than those in the TD group. During the Stroop task, there were no significant differences between these three groups in reaction time and omission errors. Both the HFA and ADHD groups showed a higher level of inactivation in the right prefrontal cortex (PFC) during the No-go blocks, relative to the TD group. However, no significant differences were found between groups in the levels of oxyhemoglobin concentration in the PFC during the Stroop task.

**Conclusion:**

Functional brain imaging using NIRS showed reduced activation in the right PFC in children with HFA or ADHD during an inhibition task, indicating that inhibitory dysfunction is a shared feature of both HFA and ADHD.

## Introduction

Autism is a disorder characterized by impairments in social interaction and communication, and a markedly restricted repertoire of social activities and interests (DSM-IV, 1994). Many studies have indicated that individuals with autism exhibit an impairment of executive function (EF). Response inhibition, an important domain of EF, reflects the ability to suppress irrelevant or interfering information or impulses. Mosconi [1] suggested that impaired response inhibition was associated with repetitive behavior in autistic patients. Bíró and Russell [2] reported significant impairments of inhibitory control in individuals with autism, and several additional studies have provided data that support this conclusion [3]–[7]. However, there is also evidence from other groups that suggests that there is no difference between autistic subjects and healthy controls with regard to inhibition measures [8]–[11]. The reasons for the above differences may have the following two aspects: first, the variation of the samples which involved age, gender, education level, IQ and the sample size [12]; second, the different tasks being used, which might involve the different components of inhibition [13], the ratios of the response to the non-response, the ratios of the neutral stimuli to the interference stimuli [14], as well as the stimulus intervals [9]. All these factors might influence the results.

Clinical observations indicate that autism and attention deficit-hyperactivity disorder (ADHD) share many similar symptoms, including inattention, hyperactivity, and poor self-control. Several studies have suggested that inhibitory dysfunction is a key neurophysiological defect of ADHD [15]–[18].

A number of groups have directly compared response inhibition between ADHD and autism groups. However, these studies have often yielded conflicting results. For instance, Ozonoff and Jensen [19] found that even though children with autism had no deficit in inhibitory control, this was not the case for children with ADHD. In contrast, later studies indicated that children with autism exhibit similar inhibition deficits to children with ADHD [6], [20]–[21]. Although the results of neuropsychological studies have demonstrated that both ADHD and autism are associated with response inhibition deficits, it is not clear whether the two kinds of disorder have the same underlying mechanism in the brain. Furthermore, the number of studies comparing brain function between these two disorders is limited. Several imaging studies have highlighted the importance of the prefrontal cortex, a region on which response inhibition is highly dependent [22]–[24]. Clearly, therefore, more attention needs to be paid to the function of the prefrontal cortex when studying brain mechanisms associated with response inhibition in ADHD and high functioning autism (HFA).

Casey [13], [25] divided inhibitory control into three subcomponents (stimulus selection, response selection and response execution) and found that each subcomponent might engage in a different cognition processing phase. The various tasks require different inhibition components. In the Stroop task, a participant needs to ignore the word's semantic meaning but just name the color of the ink in which the word is printed, given that the reading task is a highly automatic process [9] and the participant has to inhibit the meaning of the word in order to name the ink color [26]. However, in the Go/No-go task one has to inhibit the pre-potent tendency to execute a response. This inhibition process may occur only at the response-selection or execution stages [27]. Thus, there is little stimulus or response overlap that leads to the other forms of interference [14].

Besides, according to the weak central coherence hypothesis of Frith (1989) concerning the abnormal performance of individuals with autism on tasks that involve both local processing and global processing [28], the performance on the Stroop task and Go/No-go task might be inconsistent.

Functional near-infrared spectroscopy (NIRS) is an optical method for measuring changes in brain oxygenation. NIRS is noninvasive, easy to operate and conduct repeat scans, and provides data with a high temporal resolution. Thus, NIRS may be a useful approach for assessing brain function in children with developmental disorders, particularly when combined with neuropsychological tests.

Further investigation of phenotypic overlap between ADHD and autism at the behavioral and neurocognitive levels are needed for the identification of endophenotypes across diagnostic categories. The current study has attempted to compare the performances of children with autism and ADHD in tasks requiring response inhibition, as well as use NIRS to investigate any differences between these two disorders in the functioning of the prefrontal cortex during response inhibition tasks.

## Materials and Methods

### Participants

Nineteen boys with high functioning autism (HFA) (IQ≥80) and 16 boys with ADHD, recruited from the Child Mental Health Research Center of Nanjing Brain Hospital, were compared with 16 typically developing (TD) children recruited from the local community. Participants were group-matched for age, gender, full-scale IQ and handedness. All participants were right-handed, and the range of ages was 8–14 years old (see Table 1). Individuals were included in the HFA group if they met DSM-IV diagnostic criteria for autistic disorder, as well as criteria for autism according to Autism Diagnostic Interview-Revised (ADI-R). Recruitment of the ADHD group was based on both the DSM-IV diagnostic criteria for ADHD of the combined type, and the score in the Chinese version of the Swanson, Nolan, and Pelham, Version IV Sale (SNAP-IV). IQ was evaluated with the Chinese version of the Wechsler Intelligence Scale for Children-Revised (WISC-R). Typically developing children had no history of any mental or neurological disorders. Exclusion criteria for all subjects were a history of seizure or head trauma, or a diagnosis of a neurological disorder, genetic disorder, or major medical condition. All participants and their parents were informed and signed a consent form, and the study was approved by the Institutional Review Board of Nanjing Brain Hospital, affiliated to Nanjing Medical University.

**Table 1:** Subject characteristics.

	HFA (n = 19)	ADHD (n = 16)	TD (n = 16)	*F*	*P*
Age (years)	10.11**±**2.08	9.75**±**1.18	9.69**±**1.74	0.30	0.74
WISC-II FSIQ	99.26**±**9.03	103.63**±**8.13	105.63**±**13.12	1.78	0.18
The score of SNAP-IV	1.15±0.33	2.09±0.32	0.14±0.09	202.62	0.00

*Data for age and WISC-R FSIQ are presented as mean ± SD. HFA: high-functioning autism; ADHD: attention deficit hyperactivity disorder; TD: typically developing; WISC-R FSIQ: Full-Scale Intelligence Quotient measured using the Wechsler Intelligence Scale for Children-R (Chinese version). SNAP-IV: Swanson, Nolan,and Pelham, Version IV Sale(Chinese Version).*

### Go/No-go task

The Go/No-go task was a block design task comprised of two Go and two No-go blocks. Blocks were presented alternately with a 30 s resting phase. Each block included 20 trials, and in total, 80 trials were given, of which 25% were No-go (inhibition) trials. Each trial was displayed for 500 ms, with a 1000 ms inter-stimulus interval. In No-go blocks, participants were required to make a response to the letter “O”, and withhold their response when the letter “X” presented. All stimuli had the same occurrence probability of 0.5. In Go blocks, the letters “A” and “B” were shown instead of letters “O” and “X”. Participants were instructed to respond to each letter as quickly as possible, following a fixation cross. The whole experiment lasted 240 s, and the correct reaction times (RT) omission errors and commission errors (responding to a No-go) were recorded.

### Stroop task

The Stroop task was composed of two conditions, and each condition was presented twice, alternately, with a 30 s resting phase. Each block included 10 sets of stimuli, and every trial was displayed for 500 ms, with a 2500 ms inter-stimulus interval. Participants were alternately shown the letter “X” in one of four colors (red, green, yellow and blue), and black Chinese characters whose meanings represented the four colors described above, in neutral blocks. Participants were required to distinguish whether the meaning that the Chinese character represented was consistent with the color of the letter “X”, and to respond accurately, as quickly as possible.

In incongruent blocks, participants were alternately shown black Chinese characters whose meanings represented one of four colors (red, green, yellow and blue), and Chinese characters printed in those four colors. As in the neutral task, participants were instructed to make an immediate and accurate response while distinguishing whether the colored character was of the same color as described by the meaning of the black character.

The total experiment time was 240 s, and performance was measured using the correct reaction times and reaction errors in incongruent blocks.

### NIRS data acquisition

**Figure 1:**
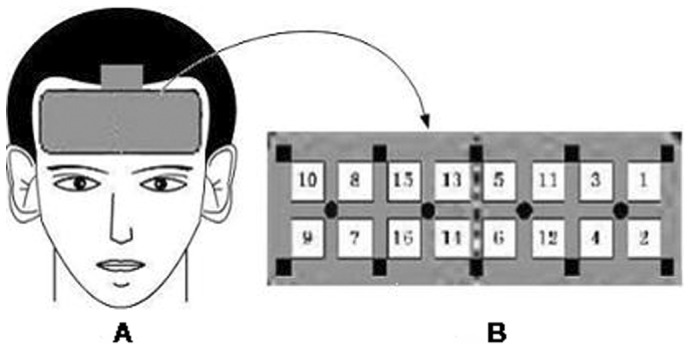
A: Schematic diagram showing the positioning of the optical probe in the head of the subject. B: NIRS channel orientation.

A 16-channel NIRS system (JH-NIRS-BR-05), developed by Huazhong University of Science and Technology, was used to measure the relative changes in oxygenated hemoglobin (oxy-Hb). This system used near infrared light at three wavelengths (735, 805, and 850 nm). In the current study, the relative concentrations of oxy-Hb were recorded at 16 measurement points in an area of 1.875×2.2 cm over the bilateral prefrontal cortex (see Fig. 1). The sampling rate used was 3 Hz. NIRS data were stored on a computer and analyzed using JH-NIRS-BR-05 software. Hemoglobin quantity was measured in arbitrary units (a.u.), meaning that the measured signal was also dependent on the path length of the near-infrared light in the brain. During the experimental session, participants were therefore required to sit in a relaxed position and avoid head movements.

### Data processing

In order to improve the signal-to-noise ratio, NIRS data were filtered with a digital bandpass set between 0.0005 and 0.02 Hz. To compensate for drift over time, a baseline correction of oxy-Hb (5 s preceding the task) was carried out, in which the mean value of the baseline was calculated and subtracted from each time point during the baseline and activation phase [29]. The average changes in oxy-Hb during each task minus the average changes in the baseline period before the task, were used for statistical analysis [30]. The mean of the values of channels 1, 2, 3, 4, 5, 6, 11 and 12 was used to represent the oxy-Hb concentration of the left prefrontal cortex; the mean of the values of channels 7, 8, 9, 10, 13, 14, 15 and 16 was used to represent the oxy-Hb concentration of the right prefrontal cortex; the mean of the values of channels 1, 3, 5, 8, 10, 11, 13, and 15 was used to represent the oxy-Hb concentration of the upper prefrontal cortex; and the mean of the values of channels 2, 4, 6, 7, 9, 12, 14 and 16 was used to represent the oxy-Hb concentration of the lower prefrontal cortex. The mean oxy-Hb value for each hemisphere, the reaction times (RT) and the reaction errors for each task were assessed by one-way analysis of variance (ANOVA). Further pair wise comparisons were performed using *t*-tests. All statistical analyses were conducted using SPSS 16.0 for Windows software, and *P*<0.05 was regarded as statistically significant.

## Results

### Performance during the Go/No-go task

The behavioral data for all subjects undertaking the Go/No-go task were considered suitable for analysis since that the error rate was not higher than 30%, as shown in Table 2. Both the HFA group and ADHD group made more commission errors than the TD group during the No-go blocks (*F* = 3.47, *P* = 0.04), whereas there was no significant difference between the HFA and ADHD groups in the number of commission errors (*P*>0.05). There were significant differences between the three groups in the reaction times during the No-go blocks (*F* = 6.92, *P* = 0.002). The results of *t*-test showed that both the ADHD and HFA groups had a longer reaction time during the inhibition task (*P*<0.05) comparing to the TD group. For the number of the omission errors, neither the HFA nor ADHD group made more than the TD group did (*P*>0.05). However, during the Go blocks there were no significant differences among the three groups in the reaction time (*F* = 1.04, *P* = 0.36).

**Table 2:** Performance data during response inhibition tasks.

Variable	HFA(n = 19)	ADHD(n = 16)	TD(n = 16)	*F*	*df*	*P*	*t* a
Go/No-go task							
Comission errors	3.95**±**2.32	3.88**±**2.50	2.25**±**1.24	3.42	2	0.04	1>3*, 2>3*
Omission errors	2.16±4.04	0.81±0.98	0.31±0.60	2.52	2	0.09	n.s
RT during No-go blocks (ms)	567.49**±**111.45	525.35**±**42.98	470.39**±**47.64	6.92	2	0.002	1>3**, 2>3**
RT during Go blocks (ms)	465.79±128.96	448.50±66.85	420.35±57.40	1.04	2	0.36	n.s
Stroop task							
Reaction errors of incongruent blocks	3.42**±**2.04	2.56**±**2.07	2.67**±**2.23	0.88	2	0.42	n.s.
RT during incongruent blocks (ms)	1740.66**±**361.05	1793.06**±**217.98	1672.95**±**312.97	0.59	2	0.56	n.s.

*Data are presented as mean ± SD. RT: reaction time.*

a *1 = HFA; 2 = ADHD; 3 = TD.*

* *P<0.05;*

** *P<0.01;*

*** *P<0.001;*

*n.s. P>0.05.*

### Performance during the Stroop task

The data for most of the subjects undertaking the Stroop task were considered suitable for analysis as the error rate was not higher than 30%, with the exception of that of one boy in the TD group. The three groups did not differ significantly in the reaction errors (*F* = 0.88, *P* = 0.42) and in the reaction time (*F* = 0.58, *P* = 0.56) during the incongruent blocks. In addition, there were no significant differences between the HFA group and TD group in both the reaction errors (*t* = 1.03, *P* = 0.31) and reaction time during incongruent blocks (*t* = 0.58, *P* = 0.57). Furthermore, the children with ADHD performed as well as the TD children during the incongruent blocks (*t* = 0.14, *P* = 0.89). Meanwhile, compared with the ADHD group, the HFA group did not made more errors in the Stroop task (*t* = 1.24, *P* = 0.23) (see Table 2).

### NIRS measurements

Analysis of the data using t-tests showed that the oxy-Hb concentration of the right prefrontal cortex in the HFA group was lower than that in the TD group during No-go blocks (−32.88×10^−4^±34×.1410^−4^ a.u. vs. 0.52×10^−4^±37.58×10^−4^ a.u.; *t* = 2.75, *P* = 0.009). Furthermore, there was a significant difference in the oxy-Hb concentration in the right prefrontal cortex between the ADHD and TD groups (−28.58×10^−4^±41.37×10^−4^ a.u. vs. 0.52×10^−4^±37.58×10^−4^ a.u.; *t* = 2.08, *P* = 0.046). However, there were no significant differences between groups in the oxy-Hb concentrations in the other three regions of the prefrontal cortex (*P*>0.05). Moreover, as shown in Table 3, the oxy-Hb concentration of the right prefrontal cortex in the ADHD group was higher than that in the TD group during the Stroop task (−7.53×10^−4^±28.53×10^−4^ a.u. vs. −36.47×10^−4^±33.01×10^−4^a.u.; *t* = 2.62, *P* = 0.01). However, there were no significant differences between HFA group and TD group in the oxy-Hb concentrations in the prefrontal cortex (*P*>0.05)

**Table 3:** The oxy-Hb concentration in the prefrontal cortex during response inhibition tasks.

Measure	HFA(n = 19)	ADHD(n = 16)	TD(n = 16)	*F*	*P*	*t* a
No-go blocks						
Left-PFC	48.80±53.08	14.00±66.10	17.00±46.39	2.12	0.13	n.s.
Right-PFC	−32.88±34.14	−28.58±41.37	0.52±37.58	3.90	0.03*	1<3**, 2<3*
Upper-PFC	0.75±14.30	0.18±13.86	4.85±17.88	0.39	0.68	n.s.
Down-PFC	1.24±15.84	1.20±12.72	−2.66±17.63	0.31	0.73	n.s.
PFC	15.92±25.32	11.90±32.24	17.53±17.57	0.21	0.82	n.s.
Go blocks						
PFC	15.91±36.09	18.54±32.34	19.57±35.61	0.05	0.95	n.s.
Left-PFC	41.53±50.68	23.81±31.03	13.68±66.46	1.33	0.27	n.s.
Right-PFC	−25.61±38.05	−5.26±32.13	5.89±38.36	3.39	0.04	1<3*
Upper-PFC	0.68±14.91	0.82±10.79	4.73±16.78	0.42	0.66	n.s.
Down-PFC	1.31±15.36	1.49±9.82	−2.28±15.33	0.39	0.68	n.s.
Incongruent blocks						
Left-PFC	−35.78±308.60	25.27±31.22	48.15±40.01	0.88	0.42	n.s.
Right-PFC	−91.40±323.65	−7.53±28.53	−36.47±33.01	0.79	0.46	n.s.
Upper-PFC	−3.24±37.72	3.78±9.35	1.00±8.95	0.37	0.70	n.s.
Down-PFC	−12.66±46.75	−1.56±10.91	0.46±9.54	0.97	0.39	n.s.
PFC	−127.17±62.80	17.74±18.12	11.68±16.32	0.79	0.46	n.s.

*Data are presented as mean ± SD. Units are ×10^−4^ a.u. PFC: prefrontal cortex.*

a *1 = HFA; 2 = ADHD; 3 = TD.*

* *P<0.05;*

** *P<0.01;*

*** *P<0.001;*

*n.s. P>0.05.*

**Figure 2:**
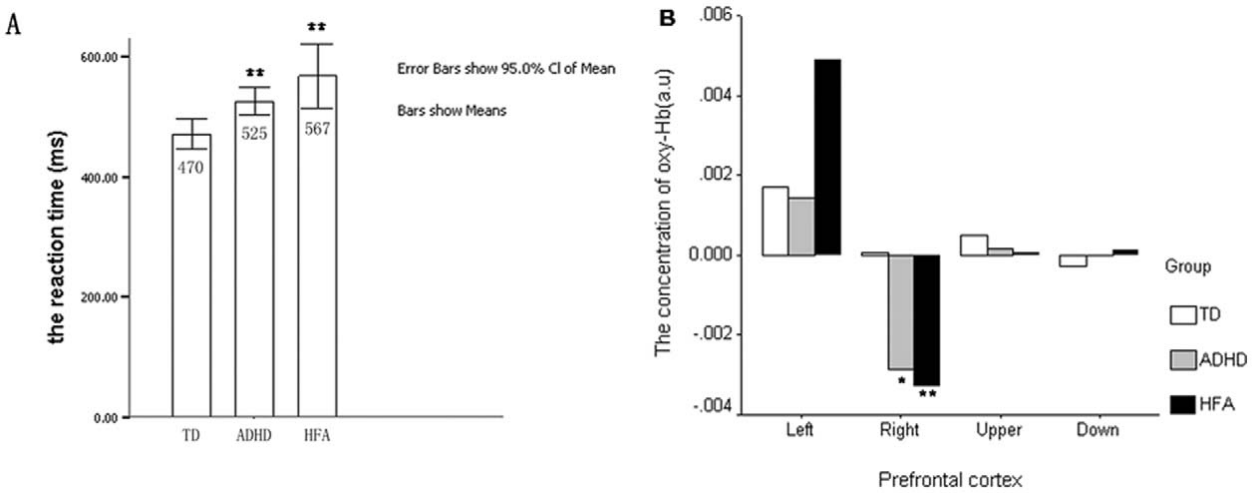
The performance of the three groups during No-go task. A: the reaction time of the No-go task; B: the oxy-Hb levels in the prefrontal cortex. Compared to TD, HFA and ADHD individuals required a longer reaction time during the No-go task. Both the reaction time and oxy-Hb level in the right prefrontal cortex are highest in HFA and lowest in TD. **: *P*<0.01; *: *P*<0.05.

**Figure 3:**
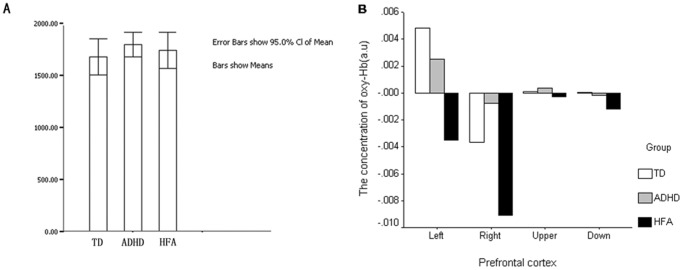
The performance of the three groups during incongruent task. A: The reaction time of incongruent task; B: the oxy-Hb levels in the prefrontal cortex. HFA and ADHD individuals had lower oxy-Hb levels in the left and HFA and ADHD individuals had lower oxy-Hb levels in the left and inferior prefrontal areas, and longer reaction times, than typically developing children.

Fig. 2 shows that, compared to typically developing children, HFA and ADHD individuals require a longer reaction time during the No-go blocks, and that both reaction time and oxy-Hb level in the right prefrontal lobe are highest in the HFA group, and lowest in the TD group. However, as may be seen in Fig. 3, we observed that during the performance of inhibitory tasks, HFA and ADHD individuals had lower oxy-Hb levels in the left and inferior prefrontal areas, and longer reaction times, than typically developing children.

## Discussion

ADHD and autism are both highly heritable neurodevelopmental disorders. There is evidence that both these conditions co-exist with a high frequency: specifically, 20–50% of children with ADHD meet the criteria for autism, while 30–80% of children with autism meet the criteria for ADHD [31]. Some family and twin studies indicate that ADHD and autism share similar familial or genetic origins, which operate across autistic characteristic and ADHD behaviors, evident both in normal variation as well as at the extremes. The present study has provided evidence that these two disorders show similar dysfunction in response inhibition, both from a behavioral perspective and from the perspective of the underlying brain mechanisms involved.

From the behavioral perspective, our data are suggestive of a dysfunction in response inhibition both in the HFA group and in the ADHD group. Compared with the TD group, children in both the HFA and ADHD groups needed more time to make a response during a No-go blocks. In addition, their difficulty might be specific to the No-go blocks, as during Go blocks no significant difference was found between each two groups. Furthermore, children in both these groups made more errors than those in the TD group during the No-go blocks. These data demonstrate that impairment of response inhibition exists in both HFA and ADHD, and this finding is in agreement with that of Christ [5]. In their study, Christ [5] gave three inhibitory tasks to 18 children with autism, to 23 siblings of children with autism, and to 25 TD children; the results showed that children with autism had a higher error rate than those of the other groups during the Go/No-go task.

However, during the Stroop task, no significant differences in error number were noted in our study between the HFA and TD children, or between the ADHD and TD children. These deferential results obtained from the Go/No-go and Stroop tasks support the viewpoint of Casey [13], and the weak central coherence hypothesis (1989). The potential superiority of children with HFA when processing the stimuli was in a less holistic way compared with TD children. Adams [26] performed a control study on 24 autistic children aged 8 to 16 years, using the classical Stroop colored letter task and the animal integrated model task, and the results showed that autistic children exhibited faster reading speed and better accuracy, but poorer reading comprehension ability. Since the classical Stroop colored letter task only requires subjects to distinguish the color of the letter but not the meaning of the character, poor reading comprehension ability in children with autism does not influence this task. Therefore, Adams concluded that the classical Stroop colored letter task may not accurately measure the inhibitory function of autistic children. These three factors may underlie the apparently conflicting observations that the inhibitory function defect in autistic children was detected in certain tasks, but not seen in some previous studies [22], [32].

From our study, we found that both reaction time and oxy-Hb level in the right prefrontal lobe are highest in the HFA group, and lowest in the TD group. Imaging studies have found that inhibitory function is correlated with the level of activation of the right prefrontal lobe [33]–[35]. The present study further supports the proposal that HFA and ADHD children have an inhibitory function defect, and this is consistent with other research conducted using brain imaging techniques. In a study by Schmitz [23], in which children with autism were asked to carry out inhibitory tasks, fMRI indicated that during the Go/No-go task, only the left lower orbital cortex, rather than the right prefrontal lobe, was activated. Subsequently, Kana et al. [24] reported similar results, also using fMRI. Smith et al. [17] also carried out a study using fMRI on ADHD children executing response inhibition tasks, and concluded that the level of activation of the prefrontal lobe was decreased. These data illustrate that it is feasible for the NIRS technique to investigate cerebral hemodynamic in autism during inhibitory tasks.

Although Table 4 showed no significant correlation between the level of inactivation in the right prefrontal and the behavioral performance during the No-go blocks, according to the Fig. 2 and statistical analysis, there was still a correlation tendency between the level of inactivation in the right prefrontal and the behavioral performance (see Fig. 2 and Fig. 3).

**Table 4:** The correlations between the level of inactivation in the right prefrontal region and the behavioural performance at the inhibition Task.

	The oxy-Hb concentration in the right prefrontal cortex	
Item	*r*	*P*
No-go blocks		
Comission errors	−0.12	0.403
Omission errors	−0.147	0.303
RT during No-go blocks (ms)	−0.083	0.568
Incongruent blocks		
Reaction errors of incongruent blocks	−0.108	0.455
RT during incongruent blocks (ms)	−0.043	0.766

RT: reaction time. * P<0.05; ** P<0.01; *** P<0.001; n.s. P>0.05.

Schroeter et al. [36] used NIRS to examine hemodynamic responses during incongruent, congruent, and neutral trials of the Stroop task in 14 adult healthy controls, and reported that the hemodynamic response was stronger bilaterally in the lateral prefrontal cortex during incongruent trials, as compared with congruent and neutral trials. This greater hemodynamic response was interpreted as stronger brain activation during incongruent trials of the Stroop task, due to interference. In the present study, the oxy-Hb level in the right prefrontal lobe did not differ among these three groups during the incongruent blocks in Stroop task. The contrasting NIRS results obtained in the Stroop and Go/No-go tasks support the view of Casey [25] that different subcomponents of execution control correspond to different nerve circuits in the upper frontal lobe, basal ganglia and thalamus. Go/No-go and Stroop tasks represent different inhibitory components and also correspond to different functional brain regions. Thus, the hemoglobin content in the frontal lobe may also differ between the various tasks.

Some limitations of this study are worth noting. First, the present study included a rather small sample size, and further research is needed to increase the sample size and strengthen the conclusions drawn. Second, because NIRS is unable detect the activities of deep sub-cortical structures where near-infrared light cannot reach, combination studies with other imaging methods are needed to further investigate possible relationships between the activity of the prefrontal cortex and the responses to stimuli.
